# Isolated radial head dislocation, a rare and easily missed injury in the presence of major distracting injuries: a case report

**DOI:** 10.1186/1752-1947-1-38

**Published:** 2007-06-29

**Authors:** Ulfin Rethnam, Rajam SU Yesupalan, Salah S Bastawrous

**Affiliations:** 1 Glan Clwyd Hospital, Rhyl, UK

## Abstract

High velocity accidents can lead to major injuries – long bone fractures, abdominal trauma, pelvic fractures and chest injuries. These injuries can act as distracting factors during the initial assessment of a polytrauma patient and innocuous but significant smaller injuries can be missed. We present a rare case of isolated anterolateral radial head dislocation in a polytrauma patient.

## Background

Isolated dislocation of the radial head in adults is rare. If neglected, these can cause restriction of forearm supination and pronation, secondary degenerative arthritis of the elbow and distal radioulnar joints. This important injury can easily be missed in the presence of major distracting injuries.

## Case presentation

A 44-year old man presented to us with a high velocity motorbike accident after a head-on collision with a truck. On arrival to the A&E, he was alert and conscious but was hypotensive and tachycardic. He complained of pain in the groin and both knees. There was no significant past history. Examination revealed extensive bruising of the pelvic region, scrotal swelling and bilateral knee effusions. Initial radiographs showed an open book type pelvic fracture but no other bony injuries were identified. Stress views of the knees in theatre revealed ligamentous laxity bilaterally. The pelvis was stabilised with an external fixator after initial resuscitation and splints applied to both knees.

**Figure 1:**
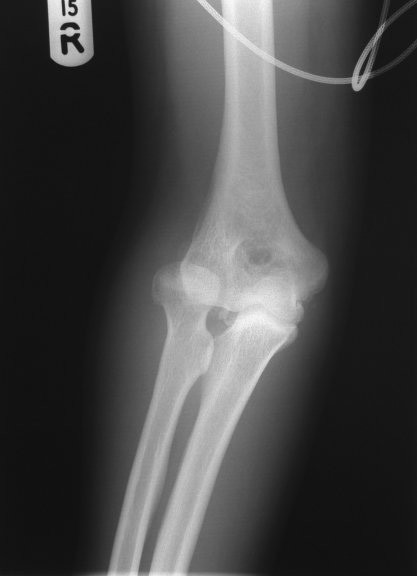
Radiograph of the elbow showing a dislocated radial head.

**Figure 2:**
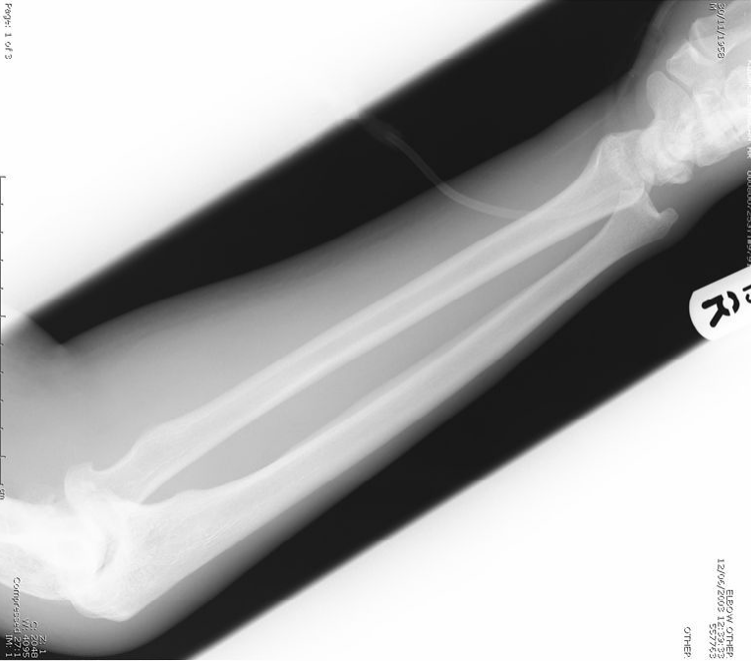
Radiograph of the elbow showing a dislocated radial head.

**Figure 3:**
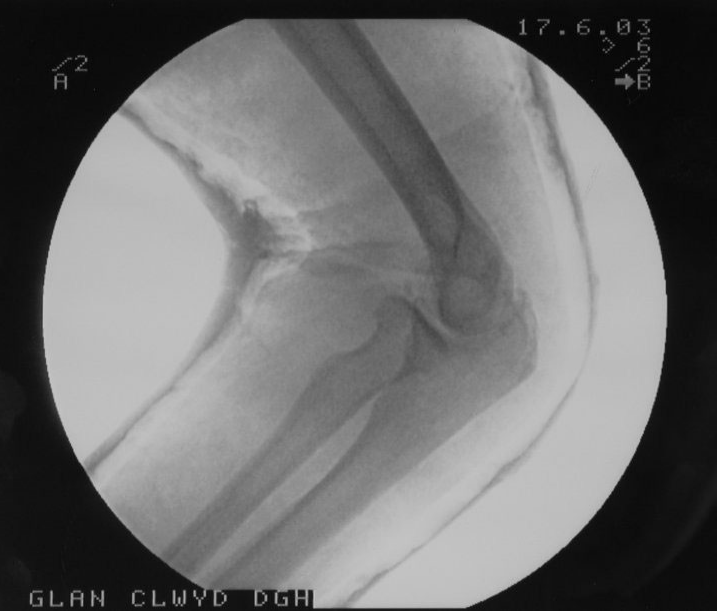
Post reduction lateral radiograph of the elbow showing the radial head in reduced position.

**Figure 4:**
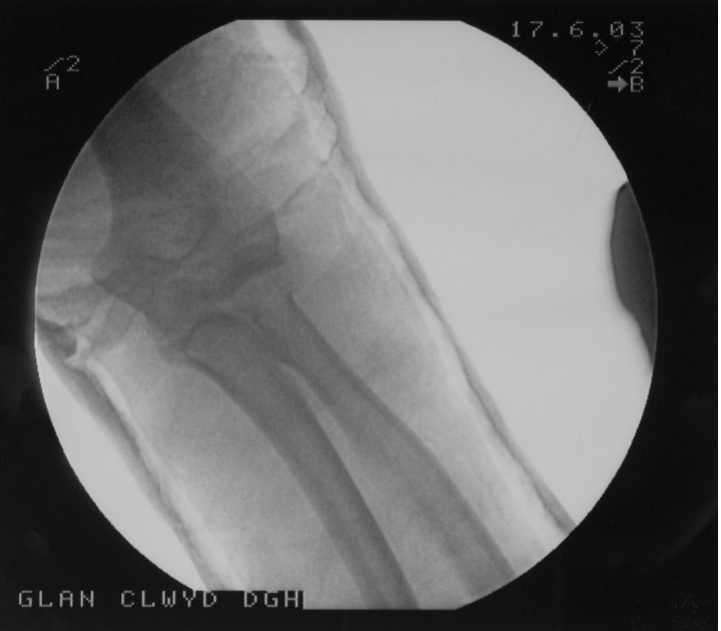
Post reduction anteroposterior radiograph of the elbow showing the radial head in reduced position.

12 hours later, the patient complained of pain in the right elbow. There was no previous history of elbow injury or arthritis. On examination, there was minimal swelling over the elbow and tenderness over the radial head. There was a flexion attitude of the right elbow. Although he had good flexion and extension of the elbow, forearm pronation and supination were restricted and painful. There was no evidence of posterior interosseus nerve palsy. Radiographs showed an anterolateral dislocation of the radial head with no associated fractures of the radius, ulna or disruption of the distal radioulnar joint. (Figure 1 &2) Closed reduction was achieved by supinating the forearm and applying pressure on the radial head following which immobilisation was done in an above elbow plaster with the forearm in supination and elbow in 90 degrees of flexion. (Figure 3 &4) The elbow was tested for stability post reduction and was found to be stable. On screening there was no evidence of a coronoid or radial head fracture. Immobilisation was continued for 3 weeks with serial radiographs done at week 1 and 2 to make sure the radial head was in reduced position. Elbow mobilisation was started after removal of the plaster under supervision of the physiotherapist. The patient was followed up at 3 and 6 months. At 6 months he had no residual pain at the elbow and movements were full elbow flexion & extension, full supination with restriction of last 10 degrees of pronation. There was no evidence of instability of the elbow.

## Discussion

Isolated dislocation of the radial head without concomitant ulnar fracture or humeroulnar subluxation in adults is a rare injury. Most cases appear to be in children. Only 20 cases have been reported in adults in the last 30 years. Most were treated conservatively with no recurrence. [1] Anterior dislocation of the radial head is even rarer with only 4 cases reported in the literature. [2] The mechanism leading to an isolated radial dislocation has been variously described. Although most authors describe an indirect mechanism, Takami et al described a direct trauma to a semiflexed elbow leading to an anterior dislocation of the radial head. [2] The postulated mechanism of injury have been described as pronation of an extended elbow [1] or traction injury to the right elbow and crush injury to the forearm [3] although Bonatus et al speculated that the injury occurred in a position of hyperextension and supination. [4] Typical clinical presentation is a maintenance of flexion and extension of the elbow following the injury but loss of supination and pronation. [1] Reduction was achieved by a pronation maneuver. [4] Most authors propose immobilization of the elbow in flexion and supination in a plaster cast [5] while Bonatus et al [4] & Negi et al [6] immobilised their cases in flexion & pronation. The period of immobilisation varied from 10 days. [1] to 4 weeks. [5] Most acute cases can be reduced closed and the functional outcome seems to be good post reduction. If missed or neglected, an open reduction has to be done with either an annular ligament reconstruction [7] or a radial head excision deemed as the procedure of choice [8].

We speculate the mechanism in our patient to be a hyperextension of the elbow with forearm in midprone position leading to an anterolateral dislocation of the radial head. The reduction was achieved in supination and immobilisation of the elbow in flexion and supination gave a favourable final outcome.

In the presence of major distracting injuries like long bone fractures, pelvic fractures, chest and abdominal injuries, an isolated radial head dislocation can be easily missed as pain is masked by the presence of major distracting injuries and flexion and extension of the elbow is normal. If supination and pronation of the forearm is not assessed, this injury can be missed resulting in degenerative arthritis of the elbow and the distal radioulnar joints.

## Conclusion

This case report has been prepared to stress the importance of a thorough secondary survey in patients with polytrauma after high impact motor vehicle accidents. A proper secondary survey in patients with major distracting injuries can prevent important injuries being missed.

## Competing interests

The author(s) declare that they have no competing interests.

## Authors' contributions

UR was involved in collecting patient details, reviewing the literature and drafted the manuscript as the main author.

RSUY was involved in reviewing the literature and proof reading of the manuscript. RSUY has approved the final manuscript.

SSB is the senior author and was responsible for final proof reading of the article.
